# Closing the Gap: Multidisciplinary Team (MDT) Staff Provision Across Ireland’s Psychiatry of Old Age (POA) Teams

**DOI:** 10.1192/bjo.2026.11388

**Published:** 2026-06-30

**Authors:** Natassha Goh, Ashok Godavarthi, Aoife O'Callaghan, Clodagh Rushe, Geraldine McCarthy

**Affiliations:** 1 Sligo Leitrim Mental Health Service, Sligo, Ireland; 2 Psychiatry of Old Age, St James's Hospital, Dublin, Ireland; 3 University of Galway, Galway, Ireland

## Abstract

**Aims:**

The older population in Ireland is expected to represent 31.6% of the population by 2057, doubling from 15.1% in 2022. As the population ages, it is important that teams are adequately staffed to deliver a high standard of care. According to Ireland's Specialist Mental Health Services Model of Care for Older People, there should be 1 POA team per 10,000 people over 65 years old, with 10 members in the MDT of various disciplines.

Our objective was to identify gaps in MDT provision in POA teams nationwide through comparing existing staffing numbers with the recommendations from the Model of Care. This data is being used to develop a strategic 5-year plan to improve staffing in POA.

**Methods:**

The College of Psychiatrists of Ireland and the National Clinical and Group Lead (NCAGL) Mental Health Office contacted Consultant POA team leads, Executive Clinical Directors and Regional Champions. Key data was collated on clinical staffing for Community Mental Health Teams (CMHTs). This data was cross-referenced, validated and compared with recommendations from the Model of Care.

**Results:**

All Regional Health Areas (RHAs) are below recommended staffing levels across all disciplines. There is a large difference in total staffing across RHAs, with the highest level of recommended staff being 61.8% in RHA F (West North West) and the lowest being 33.5% in RHA D (South West). There are also large differences between roles as well when comparing the same role across RHAs. This data was presented to key decision-makers which led to the allocation of 28 new POA posts in 2026, up from 4 posts in 2025.

**Conclusion:**

There is a significant deficit in MDT staffing nationwide. There is only 65.3% of recommended Consultant staffing in place, highlighting the need to develop new POA CMHTs. Secondly, there is significant understaffing of existing teams. Collation of staffing data is crucial in identifying staffing gaps and advocating for services.

The demographic pressures highlight the need for specialist mental health services for older people across the country. Priority must be given to POA CMHTs to adequately staff existing teams. It is also vital to plan for the development of new POA teams both in the community as well as in other integrated roles, such as in POA Acute Hospital and Nursing Home Liaison. This gap analysis identifies target areas for the 5-year plan to address unmet needs in Older Persons Mental Health.

